# ATM is the primary kinase responsible for phosphorylation of Hsp90α after ionizing radiation

**DOI:** 10.18632/oncotarget.12557

**Published:** 2016-10-11

**Authors:** Ameer L. Elaimy, Aarif Ahsan, Katherine Marsh, William B. Pratt, Dipankar Ray, Theodore S. Lawrence, Mukesh K. Nyati

**Affiliations:** ^1^ Department of Radiation Oncology, The University of Michigan Medical School, Ann Arbor, Michigan 48109, USA; ^2^ Department of Pharmacology, The University of Michigan Medical School, Ann Arbor, Michigan 48109, USA

**Keywords:** Hsp90α, ionizing radiation, γH2AX, ATM, radiosensitization

## Abstract

Heat shock protein 90 is a chaperone that plays an essential role in the stabilization of a large number of signal transduction molecules, many of which are associated with oncogenesis. An Hsp90 isoform (Hsp90α) has been shown to be selectively phosphorylated on two N-terminal threonine residues (threonine 5 and 7) and is involved in the DNA damage response and apoptosis. However, the kinase that phosphorylates Hsp90α after ionizing radiation (IR) and its role in post-radiation DNA repair remains unclear. Inasmuch as several proteins of the DNA damage response machinery are Hsp90 clients, the functional consequences of Hsp90α phosphorylation following IR have implications for the design of novel radiosensitizing agents that specifically target the Hsp90α isoform. Here we show that ATM phosphorylates Hsp90α at the T5/7 residues immediately after IR. The kinetics of Hsp90α T5/7 phosphorylation correlate with the kinetics of H2AX S139 phosphorylation (γH2AX). Although Hsp90α is located in both the cytoplasm and nucleus, only nuclear Hsp90α is phosphorylated by ATM after IR. The siRNA mediated knockdown of Hsp90α sensitizes head and neck squamous cell carcinoma cells, lung cancer cells and lung fibroblasts to IR. Furthermore, MEF cells that are Hsp90α null have reduced levels of γH2AX indicating that Hsp90α is important for the formation of γH2AX. Thus, this study provides evidence that Hsp90α is a component of the signal transduction events mediated by ATM following IR, and that Hsp90α loss decreases γH2AX levels. This work supports additional investigation into Hsp90α T5/7 phosphorylation with the goal of developing targeted radiosensitizing therapies.

## INTRODUCTION

The 90 kDa heat shock protein (Hsp90) is an abundant and essential stress protein expressed in all eukaryotic cells that aids in the post-translational folding and stabilization of a wide variety of proteins called Hsp90 “clients”, and it is an important regulator of protein homeostasis [1, 2]. The two isoforms of human Hsp90, Hsp90α (gene *Hsp90AA1*) and Hsp90β (*Hsp90AB1*), are 86 per cent homologous in amino acid sequence, are expressed from separate genes and are together referred to as Hsp90 [3]. The functions of Hsp90α and Hsp90β are thought to be largely redundant, but there are some differences. Hsp90α is highly inducible during stress, but mice without Hsp90α are apparently normal, although adult males have arrested spermatogenesis [4]. In contrast, mouse embryos lacking Hsp90β die at implantation [5]. These differences make Hsp90α an attractive pharmacological target and highlights the importance of studying the consequences of Hsp90α specific post-translational modifications. Dimerization of Hsp90 through the C-terminus is required for *in vivo* function [6].

Hsp90 is primarily located in the cytoplasm with only about 3% being in the nucleus; nevertheless, the chaperone regulates a number of nuclear events [7]. Multiple components of the DNA repair machinery are Hsp90 clients, and inhibition of Hsp90 leads to the altered localization and stabilization of repair proteins after DNA damage [8]. Three members of the phosphatidylinositol 3-kinase protein kinase-like (PIKK) family, ataxia telangiectasia mutated (ATM), ataxia telangiectasia and RAD3-related (ATR) and DNA-dependent protein kinases (DNA-PK) serve as sensors that initiate a signaling cascade resulting in DNA repair, cell cycle arrest or cell death [9, 10]. In 1989, Hsp90α was shown to be phosphorylated by DNA-PK at threonines 5 and 7 in a short segment at the N-terminus that is not present in Hsp90β, which is not phosphorylated [11].

All three of the PIKKs can phosphorylate Hsp90α, but DNA-PK is the sole PIKK responsible for Hsp90α phosphorylation during TRAIL-induced apoptosis [12], and it is the major PIKK responsible for Hsp90α phosphorylation after induction of DNA repair by short stabilized double-stranded DNA molecules (Dbait 32Hc) [13]. DNA-PK both phosphorylates Hsp90α and is an Hsp90 client [12].

Hsp90 inhibitors compromise DNA repair after radiation [14, 15], suggesting that Hsp90 plays a role in the post-radiation repair process. The inhibitors block the activity of both Hsp90α and Hsp90β, and no specific contribution of Hsp90α to the process has been defined. Here, we demonstrate that ATM is the PIKK that phosphorylates Hsp90α in response to ionizing radiation (IR) at T5/7 residues in head and neck squamous cell carcinoma cells, lung cancer cells, lung fibroblasts, ATM knockdown and reconstituted cells and mouse embryonic fibroblasts (MEF). We show that only the Hsp90α present in the nucleus is phosphorylated. The siRNA mediated knockdown of Hsp90α sensitizes cell-lines to IR, indicating a role for Hsp90α in radiation-induced DNA repair. MEF cells that are Hsp90α null have decreased phosphorylation of H2AX (γH2AX), which indicates decreased DNA repair. This indicates that Hsp90α plays a major role in post-radiation repair, but if Hsp90 is required, Hsp90β acts redundantly in the process.

## RESULTS

### Hsp90α is phosphorylated at threonine 5 and 7 following radiation-induced DNA damage

**Figure 1:**
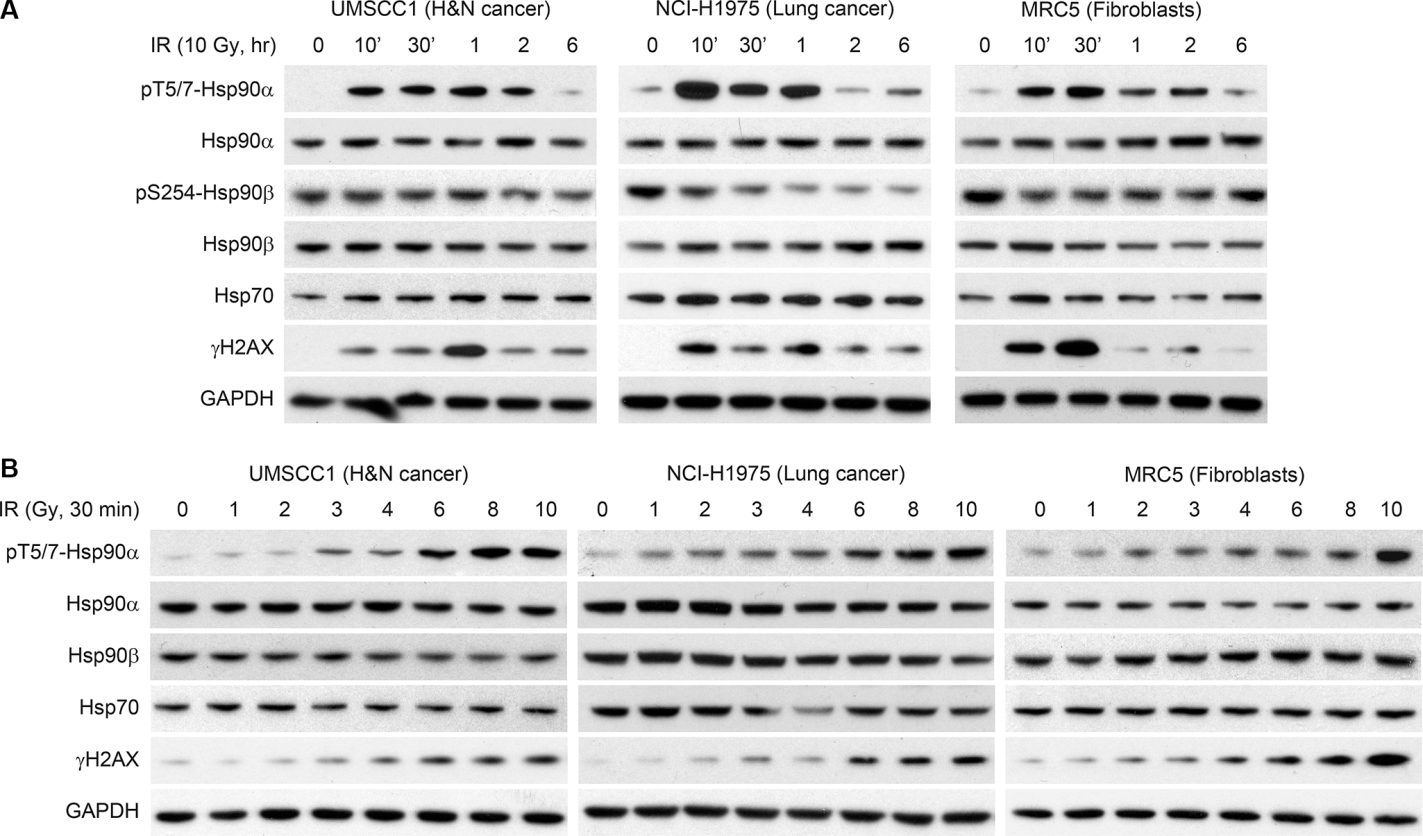
Hsp90α is phosphorylated at threonine 5 and 7 following radiation induced DNA damage (**A**) UMSCC1, NCI-H1975, and MRC5 cells were treated with 10 Gy, lysed at various time points, and immunoblotted with the indicated antibodies. (**B**) UMSCC1, NCI-H1975, and MRC5 cells were treated with various doses of ionizing radiation, lysed at the 30 minute time point, and immunoblotted with the indicated antibodies.

Figure 1 shows that Hsp90α is phosphorylated in response to IR in three cell lines (head and neck squamous cell carcinoma UMSCC1, lung cancer NCI-H1975, and lung fibroblasts MRC5). We initially studied the kinetics of Hsp90α phosphorylation in UMSCC1, NCI-H1975, and MRC5 cells treated with 10 Gy of IR in Figure 1A. In each cell line, T5/7 phosphorylation peaked early after treatment with IR and decreased to near basal levels in 6 hours. Hsp90α T5/7 phosphorylation correlated with H2AX S139 phosphorylation. Hsp90α, Hsp90β, and Hsp70 (an important co-chaperone of Hsp90) levels were unchanged over time following IR. Although Hsp90β does not contain the threonine 5 and 7 residues, we sought to determine if Hsp90β is phosphorylated at another site following IR. Serine 254 phosphorylation of Hsp90β has been reported following 5-fluorocytosine treatment in colon cancer cells [16]. As shown in Figure 1A, phospho-S254 Hsp90β was unchanged in UMSCC1 and MRC5 cells and decreased in NCI-H1975 cells. This provides additional evidence that phosphorylation following IR is Hsp90α specific. Figure 1B shows IR dose-response experiments in the same cell lines as 1A. As expected, the degree of Hsp90α phosphorylation is dose dependent and is correlated with H2AX S139 phosphorylation. Hsp90α, Hsp90β, and Hsp70 protein levels did not change upon treatment with increased doses of IR in any of these cell lines.

### ATM phosphorylates Hsp90α at threonine 5 and 7 immediately after radiation-induced DNA damage

**Figure 2:**
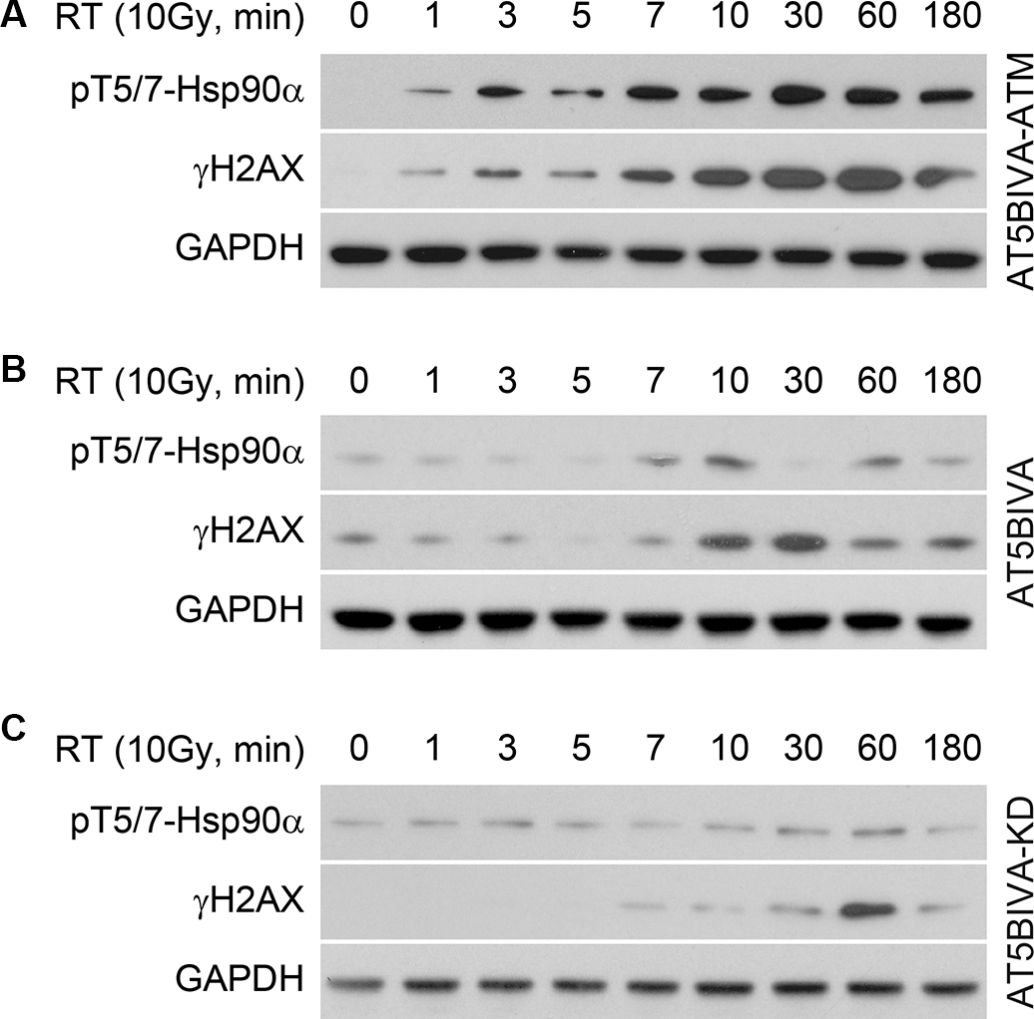
ATM phosphorylates Hsp90α at threonine 5 and 7 immediately after radiation induced DNA damage (**A**) AT5BIVA cells that have reconstituted functional ATM, (**B**) a mutation that inactivates ATM and (**C**) reconstituted kinase domain dead ATM were treated with 10 Gy, lysed at the indicated time points, and immunoblotted with anti-phospho-T5/7 Hsp90α, anti-γH2AX, and anti-GAPDH antibodies.

DNA-PK has been shown to phosphorylate Hsp90α at the threonine 5 and 7 positions following TRAIL-induced apoptosis [12] and treatment with Dbait 32Hc to induce DNA damage (T7 only) [13]. However, treatment with a specific DNA-PK inhibitor did not affect Hsp90α phosphorylation induced by IR [13]. In contrast, ATM has been implicated in post-radiation repair [17]. We investigated the role of ATM in radiation-induced phosphorylation of Hsp90α by using cells (AT5BIVA) derived from a patient with mutated ATM [18, 19]. Figure 2A shows that in AT5BIVA-ATM cells that have functional reconstituted ATM, Hsp90α is phosphorylated as early as 1 minute after IR-induced DNA damage, and Hsp90α phosphorylation is correlated with H2AX S139 phosphorylation. Figure 2B shows that in AT5BIVA cells that have an inactivating mutation in ATM (S1981), Hsp90α T5/7 phosphorylation is delayed until the 7–10 minute time point. Serine 139 phosphorylation of H2AX is also delayed. AT5BIVA cells that have reconstituted kinase domain dead ATM (AT5BIVA-KD) provide another means of studying non-functional ATM. In these cells, increases in Hsp90α threonine 5 and 7 phosphorylation are negligible and H2AX S139 phosphorylation is delayed (Figure 2C). Together, these data indicate that ATM phosphorylates Hsp90α in these cells following IR, and that a complex signal transduction network is orchestrating this cellular event.

### Phospho-T5/7 Hsp90α is located in the nucleus

**Figure 3:**
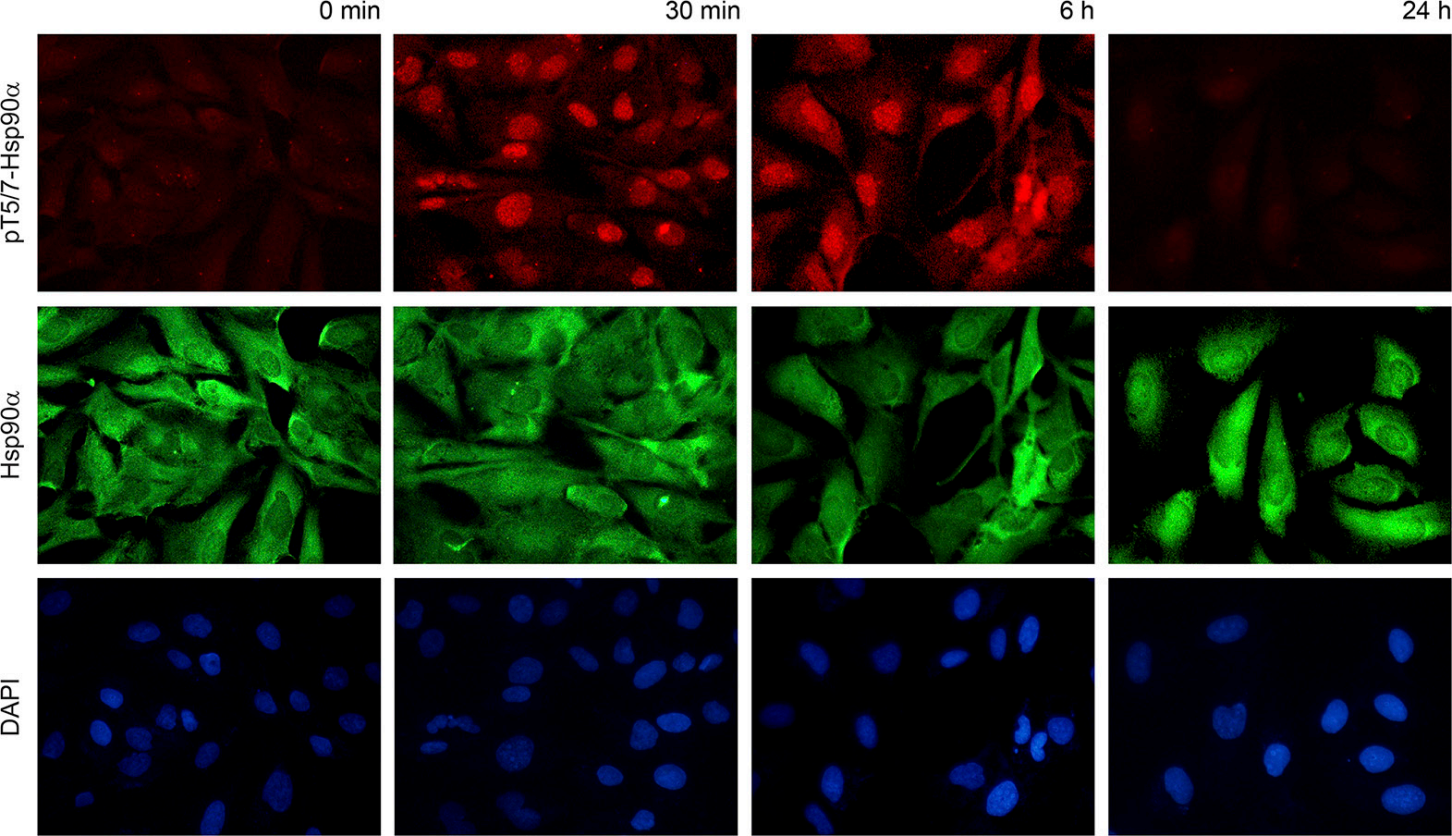
Phospho-T5/7 Hsp90α is located in the nucleus MRC5 cells were irradiated with 10 Gy and processed for immunostaining using anti phospho-T5/7 Hsp90α (red) and anti Hsp90α (green) antibodies.

To determine the cellular location of phospho-T5/7 Hsp90α we performed immunostaining in MRC5 cells. Specifically, cells were fixed at the 30 minute, 6 hour and 24 hour time points. As expected, phosphorylation was detected at 30 minutes and decreased to normal levels within 24 hours. Antibodies against phospho-T5/7 Hsp90α (red) and total Hsp90α (green) were utilized in this experiment. As shown in Figure 3, total Hsp90α is located in both the cytoplasm and the nucleus, and phospho-T5/7 Hsp90α is primarily nuclear. This provides additional evidence that the PIKK family of kinases phosphorylate the nuclear pool of Hsp90α and that phospho-T5/7 Hsp90α may be directly involved in the PIKK-mediated DNA damage signaling cascade.

### Hsp90α knockdown sensitizes cells to ionizing radiation

**Figure 4:**
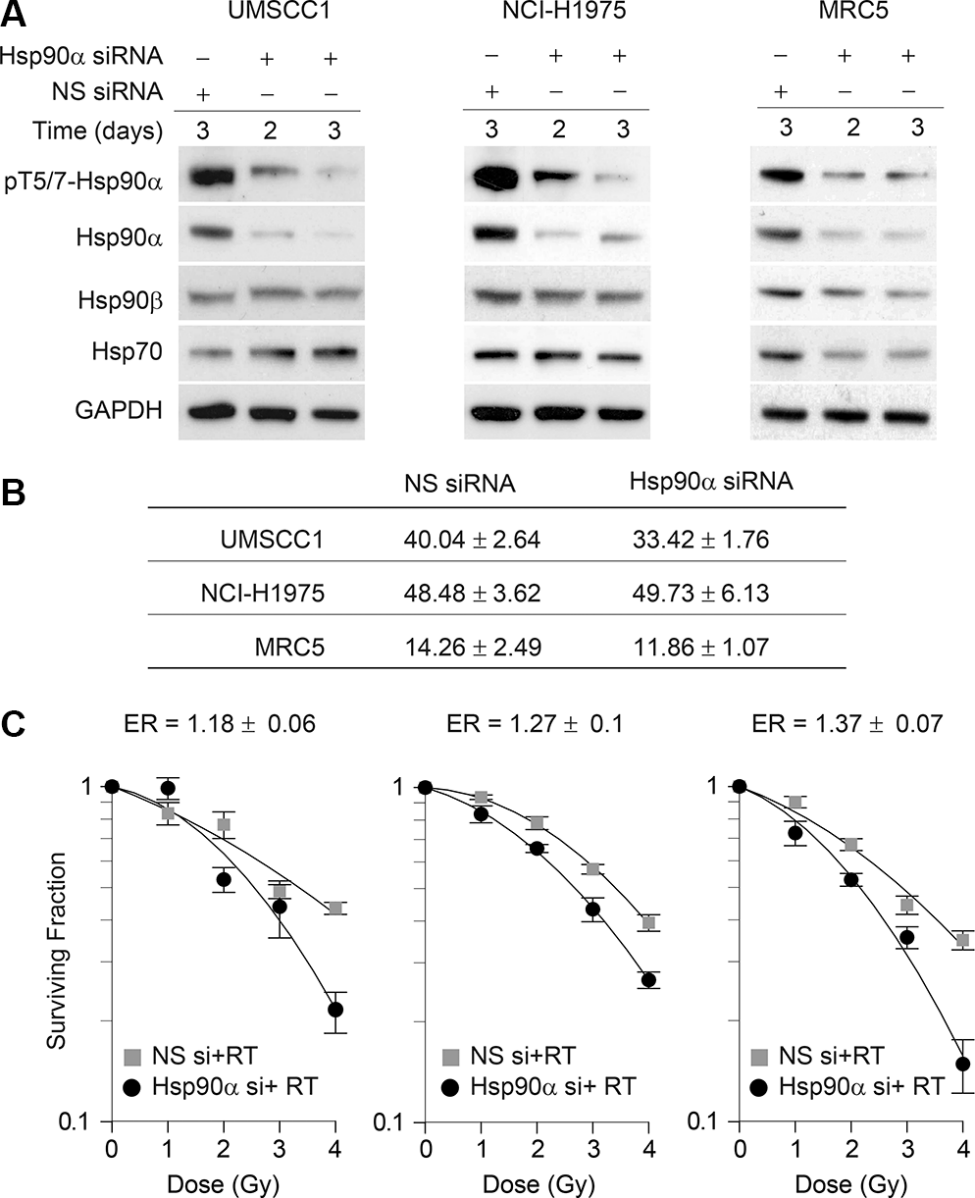
Hsp90α knockdown sensitizes cells to ionizing radiation (**A**) UMSCC1, NCI-H1975, and MRC5 cells were transfected with 50 nM non-specific siRNA or Hsp90α siRNA, lysed at the indicated time points, and immunoblotted with the indicated antibodies. (**B**) Mean plating efficiency and standard error to the mean for each cell line from three independent clonogenic survival experiments. (**C**) UMSCC1, NCI-H1975, and MRC5 cells were treated with 50 nM non-target siRNA or Hsp90α siRNA, radiated with the indicated doses of ionizing radiation 48 hours following transfection, and were processed for clonogenic survival analysis.

To determine the effects of Hsp90α deficiency on cell survival and radiosensitivity, we knocked down Hsp90α using siRNA in UMSCC1, NCI-H1975, and MRC5 cells. Figure 4A shows that the siRNA used is specific against Hsp90α and does not affect Hsp90β levels. In Figure 4B the plating efficiency and standard error of the mean are shown for each cell line. As indicated by the cell survival curves in Figure 4C, each cell line was sensitized to IR following Hsp90α knockdown. Given that nuclear Hsp90α is phosphorylated by ATM and that Hsp90α knockdown sensitizes cells to IR, this suggests that Hsp90α is directly involved in the DNA damage response.

### Hsp90α deficient cells have reduced γH2AX

**Figure 5:**
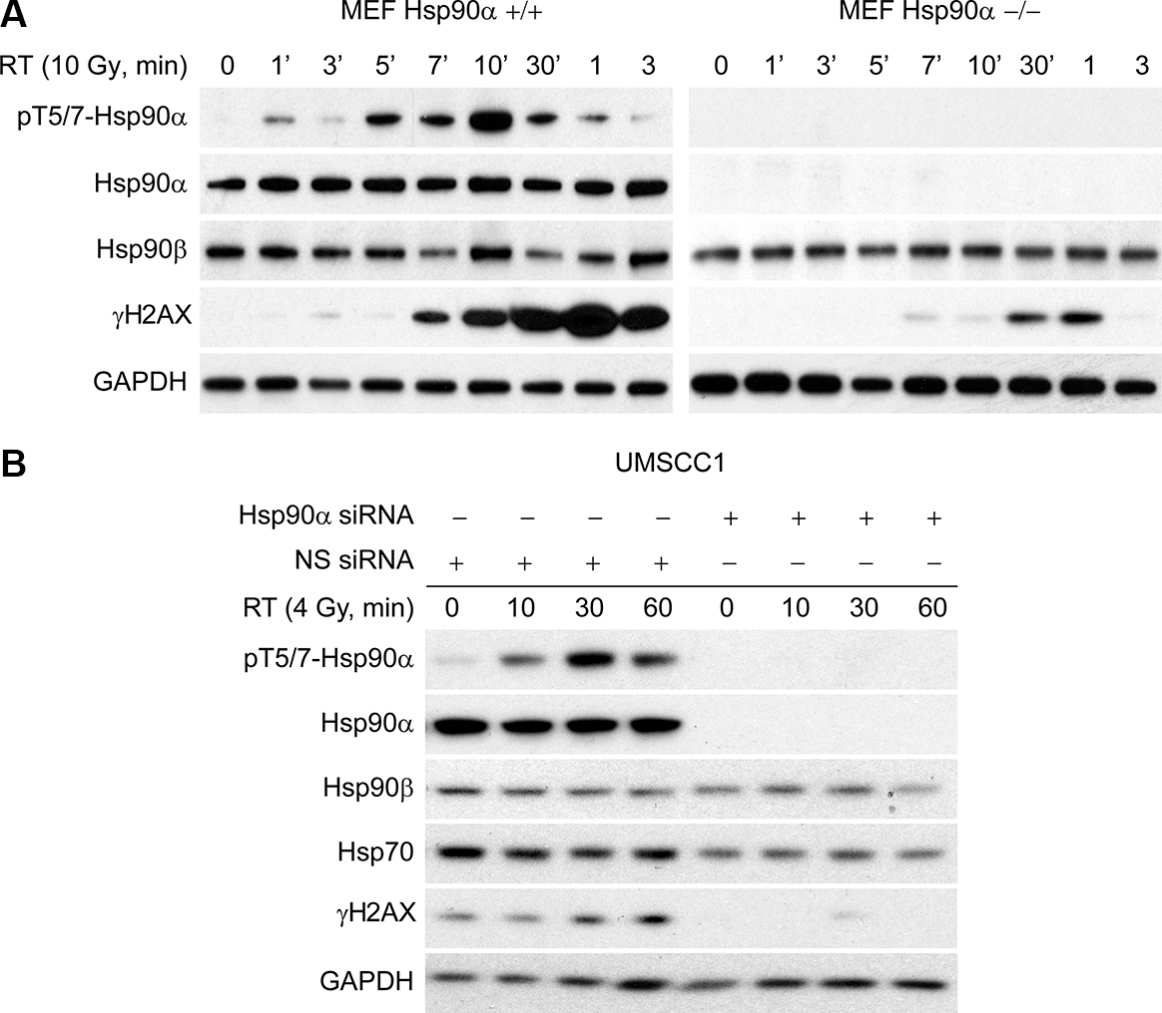
Hsp90α deficient cells have reduced γH2AX (**A**) Mouse embryonic fibroblasts that have wild-type Hsp90α and that are Hsp90α null were radiated with 10 Gy, lysed at various time points, and immunoblotted with the indicated antibodies. (**B**) UMSCC1 cells were transfected with a 50 nM concentration of non-specific siRNA or Hsp90α siRNA, irradiated with 4 Gy 48 hours following the transfection, lysed at the 10, 30, and 60 minute time points, and immunoblotted with the indicated antibodies.

Given that γH2AX is important for the formation of repair foci following DNA damage, we sought to determine if γH2AX levels are affected following IR in Hsp90α deficient cells. To accomplish this, we obtained wild-type MEF cells and MEF cells that are Hsp90α null. In Figure 5A (left), we show that MEF cells with wild-type Hsp90α have a normal DNA damage response based on the kinetics of Hsp90α threonine 5 and 7 phosphorylation and H2AX serine 139 phosphorylation. However, MEF cells that are Hsp90α null (right) have decreased H2AX serine 139 phosphorylation. Figure 5B shows that Hsp90α knockdown in UMSCC1 cells also reduces the levels of γH2AX. Together, these data show that Hsp90α is important for the formation of γH2AX after IR and provides an explanation for why loss of Hsp90α sensitizes cells to IR.

## DISCUSSION

In the present study, we show that IR induces Hsp90α threonine 5 and 7 phosphorylation by ATM and that Hsp90α deficiency reduces γH2AX levels and sensitizes cells to IR. This work provides evidence that Hsp90α is a component of the complex signaling cascade mediated by ATM and is directly involved in the DNA damage response following IR.

We demonstrate that ATM plays a major role in the threonine 5 and 7 phosphorylation of Hsp90α following IR of AT5BIVA cells. In a recent study, Solier et al. [12] reported that Hsp90α is both a chaperone and substrate of DNA-PK. Following TRAIL-induced apoptosis, the authors found that DNA-PK is the sole kinase that phosphorylates Hsp90α at the threonine 5 and 7 positions. This was accomplished by using inhibitors of ATM activity, inhibitors of DNA-PK activity, and cells that are DNA-PK null. In a separate study, Quanz et al. [13] induced DNA damage by using short, stabilized double-stranded DNA molecules (Dbait 32Hc) and also found that Hsp90α threonine 7 phosphorylation is dependent on DNA-PK. However, when using IR to induce DNA damage, the authors reported that inhibition of DNA-PK activity had little to no effect on Hsp90α phosphorylation. These data indicate that the kinase that phosphorylates Hsp90α may be dependent on the type of DNA damage or stress that is induced.

The phosphorylation of Hsp90α on threonine 5 and 7 was observed in all the cell lines used here as well as in studies previously published [12, 13]. The nuclear fraction of Hsp90α that becomes phosphorylated may be dependent on the state of the chaperone at the time of DNA damage (ATP-bound, ADP-bound, nucleotide-free) with a specific conformation favoring phosphorylation by ATM. However, at this point, the role of Hsp90α T5/7 phosphorylation in radiation response is unclear. Determining changes in chaperone activity including ATP binding activity and ability to properly fold client proteins will be crucial in understanding the functional consequences of Hsp90α T5/7 phosphorylation. Nevertheless, given that Hsp90 inhibition has been shown to compromise post-radiation DNA repair [14, 15], it is likely that Hsp90 is required for the stabilization of γH2AX. In the event that this is the case, the γH2AX that is present in Hsp90α null MEF cells (Figure 5A) suggests that Hsp90β acts redundantly to support γH2AX.

Our study supports further research into the implications of Hsp90α threonine 5 and 7 phosphorylation upon IR and other DNA damaging agents. Investigation into selective inhibitors of Hsp90α activity may lead to promising radiosensitizing agents because of its direct role in the DNA damage response.

## MATERIALS AND METHODS

### Materials

Antibodies to detect phospho-T5/7 Hsp90α, heat shock protein 70 (Hsp70), and glyceraldehyde-3-phosphate dehydrogenase (GAPDH) were purchased from Cell Signaling Technologies (Danvers, MA). Antibodies to detect Hsp90α and Hsp90β were purchased from Enzo Life Sciences (Farmingdale, NY). The antibody to detect γH2AX was acquired from Millipore (Billerica, MA) and the antibody to detect phospho-S254 Hsp90β was acquired from Abcam (Cambridge, MA). Protease inhibitor cocktail was purchased from Sigma (St. Louis, MO). Small interfering RNA (siRNA) used in this study against Hsp90α (Hsp90*AA1* Smartpool) was purchased from Dharmacon (Lafayette, CO).

### Methods

#### Cell culture

Dr. Thomas Carey (University of Michigan, Ann Arbor, MI) provided the human head and neck squamous cell carcinoma cell line, UMSCC1. Dr. J.A. Engelman (Massachusetts General Hospital, Boston, MA) provided the lung cancer cell line, NCI-H1975. Hsp90α null MEF cells and wild-type MEF cells were provided by Dr. Heiichiro Udono (Yokohama Institute, Japan). AT5BIVA, AT5BIVA kinase dead, and AT5BIVA-ATM cells were acquired from Dr. Mira Jung, (Georgetown University Medical School, Washington DC). The lung fibroblast cell line, MRC5, was acquired from the American Type Culture Collection. UMSCC1 and NCI-H1975 cells were grown in RPMI 1640 medium supplemented with 10% fetal bovine serum. MRC5 and MEF cells were grown in DMEM medium supplemented with 10% fetal bovine serum. All AT5BIVA cells were grown in RPMI 1640 medium supplemented with 20% fetal bovine serum. In all experiments performed in this study, cells were released from flasks with 0.25% trypsin and 0.2 mM EDTA and plated onto cell culture dishes the day prior to any treatment.

### Immunoblotting

Cells were scraped into a lysis buffer containing 50 mM HEPES.KOH, 150 mM NaCl, 1 mM EDTA, 2.5 mM EGTA, 1 mM NEM, 1 mM NaF, 100 μM sodium orthovanadate, 10% glycerol, 10 mM β-Glycerophosphate, 0.1% NP40, and protease inhibitor cocktail. All samples were briefly sonicated and protein lysates were centrifuged at 13,200 RPM for 5 minutes at 4°C. The supernatant was subject to protein estimation using the Bradford method at 595 nanometers. Loading buffer containing 250 mM Tris (pH 6.8), 40% Glycerol, 4% SDS, 12.5 mM EDTA, 10% β-Mercaptoethanol, and 0.08% bromophenol blue was added to each sample before it was loaded onto a 4–12% pre-cast Bis-Tris gel (Invitrogen). Protein was transferred to PVDF membranes and was blocked with 5% BSA with 1% normal goat serum in 1X tris-buffered saline and 0.1% tween 20 at room temperature for 45 minutes. Membranes were incubated with 1 μg/ml primary antibody overnight at 4°C. Following overnight incubation, membranes were washed in 1× tris-buffered saline and 0.1% tween 20 and incubated with horseradish peroxidase-conjugated secondary antibody (Cell Signaling) for 60 minutes at room temperature. Membranes were washed 3 additional times and bound antibody was detected using an enhanced chemiluminescence agent (GE Healthcare).

### Immunostaining

Immunofluorescence staining was performed using standard protocols. Briefly, MRC5 fibroblasts grown on coverslips were washed in PBS, fixed in methanol at −20°C for 15 minutes, permeabilized with 0.2% Triton-x-100 on ice for 5 minutes, blocked for 60 minutes, and incubated in primary antibody at 4°C overnight. The primary antibody dilution for phospho- Hsp90α and Hsp90α was 1:100 in 5% BSA. The following morning the coverslips were washed using PBS, incubated with the fluorescence-conjugated secondary antibody for 60 minutes, washed again using PBS, and prepared with a coverslip using a drop of ProLong Gold anti-fade reagent with 4, 6-diamidino-2-phenylindole (Molecular Probes). Fluorescence images were obtained using a DS-Fi1 (Nikon, Melville, NY) camera fitted on an Olympus 1×-71 microscope.

### siRNA knockdown of Hsp90α and transfection

Hsp90α knockdown was accomplished in UMSCC1, NCI-H1975, and MRC5 cells by using the lipofectamine-based protocol (Invitrogen) to transfect 50 nM of Hsp90α siRNA or non-specific siRNA in Opti-mem (Gibco). The Opti-mem was replaced with cell culture media 24 hours following transfection, and the cells were irradiated and processed for further analysis 48 hours after transfection.

### Clonogenic cell survival assay

Clonogenic survival assays were performed by plating 75,000 cells in 60 mm cell culture dishes. Following the transfection (48 hours for Hsp90a knockdown) cells were radiated, trypsinized, and plated in 6-well cell culture dishes in triplicate. The UMSCC1, NCI-H1975, and MRC5 cells were plated based on a predetermined plating efficiency in 6-well cell culture dishes. Six to nine days later, cell colonies were fixed with acetic acid/methanol (1:7 ratio) and stained with a crystal violet (0.5%, w/v) solution. A stereomicroscope was used to count cell colonies. The fraction of cell colonies surviving each treatment was normalized to the survival of the control cells in each experiment. The cell survival enhancement ratio was calculated as the ratio of the mean inactivation dose in control cells divided by the mean inactivation dose in treated cells [20]. Experiments in this study were conducted three independent times.
